# Effectiveness of blended learning basic life support module on knowledge and skills: A systematic review of randomized controlled trials

**DOI:** 10.1016/j.heliyon.2023.e21680

**Published:** 2023-11-02

**Authors:** Ashraf Jehad Abuejheisheh, Jafar Alasad Alshraideh, Nawwaf Amro, Salam Bani Hani, Muhamamd Waleed Darawad

**Affiliations:** a Faculty of Health Professions, Al-Quds University-Jerusalem, Palestine; b School of Nursing, Adult Health Department, Irbid National University, Irbid, Jordan; c School of Nursing, The University of Jordan, Jordan; d Modern University College- Ramallah, Palestine

**Keywords:** “Basic life support”, “Blended learning”, “Traditional methods”, “Knowledge”, “Skills”, “Nursing, “Laypersons”

## Abstract

**Aim:**

To examine the effectiveness of the BLS blended learning module on knowledge and skills of BLS compared to the traditional module.

**Method:**

Preferred Reporting Items for Systematic Review and Meta-Analysis (PRISMA) guidelines were utilized using key words to searched PubMed, Web of Science, and Cochrane Library for the studies published between January 2018 to May 2022. **The risk of bias** was assessed utilizing the Joanna Briggs Institute (JBI) critical appraisal checklist. Two reviewers separately extracted data from the included trials using a standardized data extraction form.

**Results:**

From 400 articles retrieved by the initial search, 11 studies were found to be eligible. Most studies’ participants were laypersons (80.9 %), and the rest were either nursing (12.6 %) or medical students (6.5 %). The review shows superiority of utilizing the blended strategy in applying the BLS module in skills and knowledge retention, rather than using the traditional learning, which could improve the quality and outcomes of patients.

**Conclusions:**

Blended learning is effective in teaching BLS like the traditional face-to-face method, but more advantages of the blended learning module include improvement in retaining knowledge, skills acquisition, patient outcomes, and cost saving. The COVID-19 pandemic made blended learning crucial and using this method in BLS was effective and efficient. Future research to assess the effectiveness of blended learning on patient outcomes is recommended.

## 1 Introduction

Cardiovascular disease (CVD) is the leading cause of death worldwide with a prevalence of annual 17.3 million deaths [1]. In the United States (US), around 209,000 in-hospital cardiac arrests (IHCA) occurred in 2016 [2]. In China, more than 230 million people with CVD, and 550,000 individuals experience cardiac arrest (CA) every year [3]. Moreover, in European countries, IHCA is a major cause of death and causes a tremendous burden on their healthcare systems and resources [4] where as In Australia, the incidence of out-of-hospital cardiac arrest (OHCA) was approximately 25,000 annually. The prevalence of IHCA has been estimated as 1–5/1000 hospital admissions in developed countries [5]. Furthermore, in the Mediterranean region, there are a high number of CVD and CA cases as well. For instance, in the Islamic Republic of Iran, 50 % of all deaths each year and 79 % of deaths related to chronic diseases are attributed to CVDs [6].

The high numbers of CA cases require training programs such as Basic Life Support (BLS) to deal with such cases and increase the survival rate [7]. The first line response to CA is BLS and/or Advanced Life Support (ALS). Healthcare providers working in Emergency Departments (EDs), Intensive care units (ICU), and across healthcare facilities where in-hospital resuscitation, and out of hospital are required to gain highly qualified knowledge and skills to perform BLS in a way to decrease the deaths resulting from CA cases [7].

Basic life support training is one of the crucial strategies that could increase the chances of CA victims’ survival rate by healthcare providers [7]. However, some published studies discussed the loss of BLS skills and knowledge over time [[8], [9], [10]]. Recent studies showed that the survival rates remained low at 11.4 % for outpatient heart attacks and 23.8 % for in-hospital heart attacks, even when CPR procedures were applied [11]. In the US, approximately 209,000 IHCA occur annually, with less than a 25 % survival rate [2].

Basic life support courses have many modules to be taught. The first method is traditional face-to-face instruction. Another option is the blended approach, which is separated into two sections: a face-to-face component and an online section [7]. The traditional approach, also known as offline, is designed to provide in-person instruction for students seeking a BLS certificate. The goal of blended learning (BL), which combines offline and online instruction, is to provide online sessions before bringing a candidate in for in-person instruction and training [12,13].

Blended learning has become necessary due to technological advancements and the demands of the Covid-19 condition, which requires protocols to maintain social distance. When comparing BL to complete offline and full online methods, several studies have demonstrated that BL can increase learning results or better than traditional or entirely online learning, while success rates vary by field [14]. A study predicted that by enhancing student understanding of CA first aid through BLS training, the survival rate in the community would improve [15].

Evidence showed blended learning for BLS has some pros and cons. A randomized control trial study conducted in Belgium to investigate the effect use of different BLS blended learning modules shows many advantages. For instance, the participants who were bachelor's students demonstrated a high level of knowledge and skills of the main BLS principles. An overall median BLS score of 83 % was high when compared to other research using similar scales for BLS assessment on similar test populations. Furthermore, participants' engagement was noticed to be high compared to the face-to-face module of teaching [27]. In their cost analysis study, blended learning was cost saving than face-to-face [33]. On the other hand, blended learning needs high-quality internet and technology to implement it, thus in developing and poor countries this module may be challenging. Eventually, it is imperative to investigate a systematic review to explore this phenomenon and examine the effectiveness of blending learning in more depth with strong evidence such as randomized control trials (RCTs) or systematic review of RCTs.

Ultimately, the improved BLS competency among nurses and healthcare providers may be reflected positively on the quality of care for patients and their families and improve the quality of the provided care physically and psychologically. Physical benefits mean a decrease in the mortality rates from CA cases, and psychologically through dealing appropriately in terms of either client's death or survival since these events are not easy to accept. However, literature has contradicted results regarding face-to-face versus BL regarding BLS education. Therefore, this systematic review (SR) aimed to investigate the impact of BL on the knowledge and abilities of BLS in comparison to traditional training. Specifically, this systematic review is trying to answer two main questions; is using BL to teach BLS effective in terms of knowledge and skills? is using BL to teach BLS superior to traditional education in terms of knowledge and skills?

## 2 Methods

The Preferred Reporting Items for Systematic Reviews and Meta-Analyses (PRISMA) were followed during the planning, execution, and reporting stages of this systematic review [16]. There was no need for ethical review or patient consent because this was a systematic evaluation of previously published research.

### 2.1 Search strategy

The investigators searched in three databases that yielded indexed peer-reviewed articles {PubMed, Web of Science, Scopus}: PubMed through Medline, Cochrane Library, and Web of Science. Keywords were employed in the search. The phrases "basic life support" or "BLS" or "cardiopulmonary resuscitation" and "blended learning" or "traditional learning," as well as "knowledge," "skills," and "nursing," "laypeople," and "physicians," were added. Two researchers independently reviewed the titles and abstracts of the published publications as well as the full-text studies. A third author made the decision, when conflicting views persist. In addition, from the reference lists, the researchers examined the most relevant studies to find any missing studies that were not accessed. We searched for published articles from 2018 to May 5, 2022 regarding BL of BLS module as an educational approach.

### 2.2 Selection criteria

The search process focused on randomized control trials (RCTs) that included BL as the intervention group and usual or traditional education of BLS as the control group; healthcare providers and non-healthcare providers; containing at least one of both outcomes (knowledge or skills) and written in the English language. Additionally, studies of ay non-RCTs such as quasi-experimental, cohort, observational qualitative studies, published in a non-English language, and which have patients with cognitive impairment, unpublished articles grey literature, student's dissertations, thesis book chapters and encyclopedias, were excluded.

### 2.3 Risk of bias within studies and certainty of evidence

The risk bias assessments are summarized in Table 1. All reviewers determined the risk of bias by utilized Joanna Briggs Institute (JBI) [17,18]. If the scores of appraisals were different, an approval was completed by referral to all reviewers. The included studies randomly assigned the participants using the random assignment. All the studies did not, however, follow the double-blinded approach in their design, which is thought to be a source of bias in their results.

**Table 1:** JBI critical appraisal checklist for randomized controlled trails (n = 11).

	Domain	(Leszczyński, Charuta et al., 2018)	(Castillo et al., 2018)	Leszczyński, Gotlib et al. (2018)	(Madou & Iserbyt, 2020)	(Lehmann et al., 2019)	(Moon & Hyun, 2019)	(Bylow et al., 2019)	(Birkun et al., 2019)	(Chien et al., 2020)	(Kim et al., 2021)	(Kim et al., 2021)
1	**Was true randomization used for assignment of participants to treatment groups?**	Yes	Yes	Yes	Yes	Yes	Yes	Yes	Yes	Yes	Yes	Yes
2	**Was allocation to treatment groups concealed?**	Yes	NA	No	No	Yes	No	No	Yes	No	No	No
3	**Were treatment groups similar at the baseline?**	Yes	Yes	Yes	Yes	No	Yes	No	Yes	Yes	Yes	Yes
4	**Were participants blind to treatment assignment?**	Yes	NA	NA	No	Yes	Yes	No	Yes	No	No	No
5	**Were those delivering treatment blind to treatment assignment?**	Yes	No	No	No	No	No	Yes	NA	No	No	No
6	**Were outcomes assessors blind to treatment assignment?**	NA	No	No	No	No	No	No	No	No	No	No
7	**Were treatment groups treated identically other than the intervention of interest?**	Yes	Yes	Yes	Yes	No	Yes	No	No	Yes	No	Yes
8	**Was follow up complete and if not, were differences between groups in terms of their follow up adequately described and analyzed?**	Yes	Yes	Yes	Yes	Yes	Yes	Yes	Yes	Yes	Yes	Yes
9	**Were participants analyzed in the groups to which they were randomized?**	Yes	Yes	Yes	Yes	Yes	Yes	Yes	Yes	Yes	Yes	Yes
10	**Were outcomes measured in the same way for treatment groups?**	Yes	Yes	Yes	Yes	Yes	Yes	No	Yes	Yes	Yes	Yes
11	**Were outcomes measured in a reliable way?**	Yes	Yes	Yes	Yes	Yes	Yes	Yes	Yes	Yes	Yes	Yes
12	**Was appropriate statistical analysis used?**	Yes	Yes	Yes	Yes	Yes	Yes	Yes	Yes	Yes	Yes	Yes
13	**Was the trial design appropriate, and any deviations from the standard RCT design (individual randomization, parallel groups) accounted for in the** **conduct and analysis of the trial?**	Yes	Yes	Yes	Yes	Yes	Yes	Yes	Yes	Yes	Yes	Yes

### 2.4 Data extraction

Using a standardized data extraction form [19], two reviewers separately extracted data from the included trials. Authors, year, setting, country, design, subjects, sample size, intervention, comparator, outcomes, measurements, and trial registration were all retrieved from eligible studies. The type of training, digital component, training content, examiner, and assessment technique were retrieved. After the extraction process, the two reviewers met to double-check the results. When inconsistencies persist, a third reviewer examined full-text publications for verification**.**

## 3 Results

### 3.1 Study selection

**Fig. 1:**
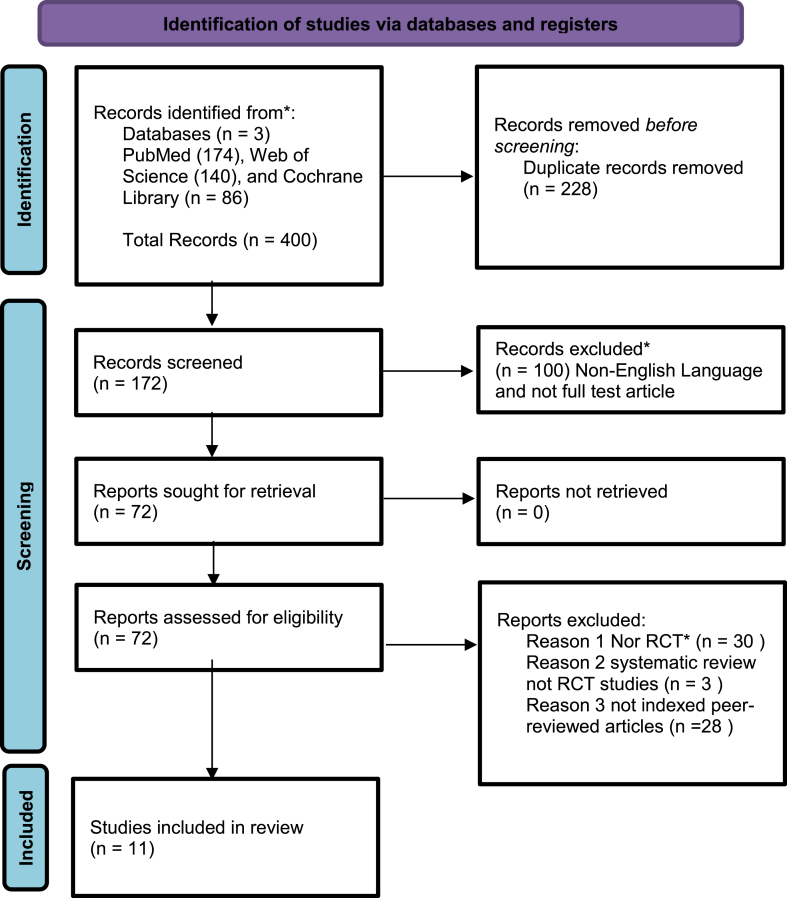
PRISMA 2020 flow diagram for new systematic reviews. *RCT = Randomized Controlled Trial.

Among the three databases, 400 studies were found in the initial search. Among 400 studies, 389 were excluded due to two main reasons, the first one due to duplicate records (228 studies), and the second one other reason such as non-English language and not full-test article (100 studies). We screened 72 studies and excluded 61 for other reasons when reading the articles such as not randomized controlled trials, being systematic reviews not RCT, and not indexed peer-reviewed articles. We used the parallel check of the EndNote function to check for the duplicate after downloading all the citations from different searched databases. Finally, 11 RCTs were found and included in the analysis. The flow diagram depicts the process of study selection in Fig. 1.

### 3.2 Study characteristics

Of the 11 included RCTs, one study was conducted in 2021, two in 2020, five in 2019, and three in 2018. Regarding the country, three studies conducted in Korea; two in Poland; and one in the following countries, Taiwan, Russia, Sweden, Germany, Belgium, and Spain. The vast majority were laypersons (80.9 %), and the rest were either nursing students (12.6 %) or medical students (6.5 %). Most of the studies were conducted in a classroom, and only one was conducted in a CPR training center. Details in Table 2.

**Table 2:** Characteristics of the studies included in the SR.

Authors/Study year	Country	Type of people/Sample size	Setting	Duration of blended intervention	Outcomes
Han et al., 2021 {22}	Korea	Lay persons n = 62 Control = 31 Intervention = 31	Training center	Distance learning (DL). The total training time was 60 min	A significant change was found in the mean compression depth (before: 47 mm vs. after: 49 mm, p < 0.001) in DL group. While no significant change in Conventional Learning CL group.
Kim et al., 2021 {23}	Korea	Nursing students. n = 64 Control = 33 Intervention = 31	Classroom setting	Non-contact CPR training using smartphone 40-min theoretical online lecture session and an 80-min non-contact practice session	The noncontact CPR training significantly increased the accuracy of chest compression (P < 0.001) and mouth to-mouth ventilation (P < 0.001), and the overall performance ability (P < 0.001). The noncontact CPR training significantly increased the accuracy of chest compression in the experimental group from 52.06 before training to 92.65 right after training and slightly decreased to 90.39 4 weeks later (P < 0.001). The noncontact CPR training significantly increased the accuracy of mouth to-mouth ventilation from 48.81 at baseline to 89.10 at right after and slightly decreased to 85.26 at 4 weeks later (P < 0.001). The noncontact CPR training significantly increased the overall performance ability from 23.10 at baseline to 37.23 at right after the training and slightly decreased to 34.87 at 4 weeks later (P < 0.001).
Chien et al., 2020 {30}	Taiwan	Lay persons n = 832 Control = 416 Intervention = 416	classroom setting	Either a traditional or blended (18-min e-learning plus 30-min hands-on) compression-only CPR training program. Compared traditional method 90-min total divided by (30 min knowledge and 60 hands on practice)	Among CPR section, AED section, compression rate, compression depth and full chest recoil. The compression depth was M = 5.21, SD = 0.66 cm in the blended group and M = 5.24, SD = 0.63 cm in the traditional group (p = 0.006). However, no significant difference was found between other skills.
Birkun et al., 2019 {29}	Russian	Nursing students and nonmedical university students. n = 94 Control = 55 Intervention = 39	Classroom	1 h for online learning and 3 h for classroom learning.	The assessment of the CPR quality in the simulation scenario revealed no significant differences between groups, excepting higher rate of chest compressions in the blended training group (116.0 vs. 109.4, р<0.01).
Bylow et al., 2019 {20}	Sweden	Laymen n = 2529 Control = 1293 Intervention = 1239	Classroom	90–120 min for BLS training and 30 min or more for web-based education	The BLS + WEB education was more effective and the participants obtained a significantly higher total score for adherence to practical skills in the BLS algorithm six months after training compared with the BLS without the web-based education. Check responsiveness, call 112, open airway, ask for an AED and attach electrode pads correctly in areas were performed significantly more effectively in the BLS + WEB group than in the BLS group, six months after training
Moon & Hyun, 2019 {28}	Korea	Nursing students n = 120 Control = 60 Intervention = 60	Classroom	The blended learning CPR education program 230 min consists of program orientation, watched a videos, lectures. The control group had only the 90-min lecture	The intervention group's CPR knowledge score significantly increased (p=<0.001). The intervention group's CPR attitude score significantly increased (p=<0.001). The intervention group's CPR self-efficacy score significantly increased (p = 0.001).
Lehmann et al., 2019 {24}	Germany	medical students n = 103 divided to three groups Video group (n = 37). Virtual Patients (VP) with animation (n = 35). Virtual patients static media (N = 31).	Classroom	All participants received a manual explaining the algorithm and steps of PBLS including flow charts. For self-instruction, a preparatory phase of 30 min was given with either VP work-up or watching the instructional videos	Statistically significant differences were found between groups for adherence to temporal demands p < 0.001. Post hoc tests revealed superior and highly significant differences for both groups using animated media (group Vid and VP animation) compared with group VP static media. The post hoc tests showed a significant superior performance by the VP group using animated media (group VP animation) compared with the VP group that used only static media (group VP static media). The overall competency rating of the three study groups differed highly significant p = 0.001. The VP group using animated media (group VP animation) received distinctly more ‘competent’ ratings than the video group
Madou & Iserbyt, 2020 {27}	Belgium	Bachelor students n = 127 divided into four groups Group 1 = 35 Group 2 = 31 Group 3 = 32 Group 4 = 29	Classroom	A mastery learning (ML) versus a self-directed learning (SDL) blended. In all blends, an online learning module was available for one week prior to a face-to-face learning component of which the duration was 45 min.	Regarding students' CPR and BLS performance included chest compression rate and depth, no significant differences between groups were found. The total BLS scores were not significantly different between groups
Leszczyński, Gotlib et al., 2018){26}	Poland	Students of emergency medicine n = 106 Students of emergency medicine n = 72 and nursing n = 34.	Classroom	The online course consists of non-animated presentation, video and interactive video.	A significant increase in knowledge in the experimental group which used an interactive video was observed (p = 0.04). Moreover, the number of replays of learning material was the highest in interactive video than other (2.09 ± 2.48) (p = 0.05)
Castillo et al., 2018 {21}	Spain	First-year students of medicine and nursing n = 129 Control = 68 Intervention = 61	Classroom	Control group (face-to-face training) and an experimental group (blended training). Received an official BLS-AED face-to-face course with 6 h. 45 min of instructor time were introduced to clarify any doubts and complete some practical cases	Immediately after the course, there were no statistically significant differences in knowledge between the two groups. The median score of practical evaluation assessed by the instructor was significantly better in the experimental group (P = 0.02). No differences between groups were found when using a high-fidelity manikin to evaluate chest compressions and lung inflations. At six months, the scores in knowledge and skill performance were significantly lower compared to the evaluations at the end of the instruction, but they remained still higher compared to baseline. The experimental group had higher scores in practical skills evaluated by the instructor than the control group (P = 0.01).
Leszczyński, Charuta et al., 2018 {25}	Poland	Students n = 65 Divided into three groups Group A = 20 Group B = 20 Group C = 23	College of Health Sciences	Three different forms of e-learning course included Course A – textual-graphical materials. Course B – audiovisual material. Course C – audiovisual material. Duration of intervention not available	The level of satisfaction in all the groups surveyed was comparable. The growth rate for knowledge was the highest in audiovisual material than other material in the second and third survey, respectively (30 and 90 days after the end of the course). A significant difference in favor of audiovisual material was demonstrated one month after finishing classes.

### 3.3 Effectiveness of BLS blended learning

Among the 11 selected RCTs, nine studies resulted in the superiority of BL compared to the traditional method on at least one aspect like knowledge or skills or other variables [[20], [21], [22], [23], [24], [25], [26], [27], [28]]. In addition, some aspects of them stated that utilizing BL is superior to the traditional method in terms of acquiring and retaining skills and knowledge over time. Two of the mentioned RCTs [26,27] pointed out that knowledge and skills were improved in different kinds of BL types despite the duration and content. Interactive audiovisual material in BL was the best option [25]. However, only two studies revealed that the outcome of the RCTs that used BL to teach BLS courses was effective as the traditional method (face-to-face). Furthermore, there was no statistically significant difference between both modules concerning knowledge and skills [29,30].

Some studies showed superiority of blended BLS over other methods. For instance, in terms of skills that depth and rate of chest compression and checking victim responsiveness were more effective in BL than the traditional method. A significant change was found in the mean compression depth (before: 47 mm vs. after 49 mm, p < 0.001) in the BL group. While no significant change in the Conventional Learning group [22]. Overall performance ability and skills were improved better in a blended group compared to the traditional group [23]. In addition, check responsiveness, open airway, ask for an Automated External Defibrillator (AED) and attach electrode pads correctly in areas were performed significantly more effectively in the BL [20,23]. Furthermore, knowledge of calling the emergency number and knowing the cases that need initiation of CPR were significantly better in experimental group than the control group. Regarding the knowledge of BLS, some studies revealed the better scores of knowledge in the experimental groups of BL compared to the traditional method [21,26,28]. All the included RCTs said that there was no any statistically significant difference in regards to the demographics such as age, gender and previous experience about CRP. Finally, in term of ventilation skills and knowledge, this aspect was not examined [30].

## 4 Discussion

Although the findings of this review suggest that BL is effective in teaching BLS as a traditional face-to-face method for educational outcomes, some aspects are better in the BL. These aspects include but are not limited to knowledge, skills acquisition, and cost saving. These findings are congruent with much-published evidence. For instance, a systematic review to see the effectiveness of BL in BLS training among nursing students revealed that BL is superior to face-to-face BLS learning in terms of knowledge, attitude, skills, self-efficacy, problem-solving abilities, and willingness to perform resuscitation [31]. Furthermore, a systematic review was conducted to evaluate the effectiveness of educational and resource outcomes of blended compared to non-BL approaches for participants undertaking accredited life support courses including BLS, ALS, and Advance Trauma Life Support (ATLS), authors stated that using BL module is at least as effective as non-BL for accredited BLS, ALS, and ATLS courses regarding knowledge, skills, and attitude [32].

The beneficial effect of BL is not limited to knowledge, skills, and attitudes but also extends to lowering cost of such courses and reducing the expense on candidates, providers, and suppliers of BLS. For example, a cost minimization analysis was performed in a study to compare the cost of both BLS blended and face-to-face, the authors pointed out that the savings of a course in BLS-AED based on the blended-learning methodology calculated for a total of 160 university nursing and medical students were € 2328.8 for the first year of its implementation and € 9048.8 for its second edition compared with the same course using a face-to-face methodology [33]. Moreover, the annual projected costs for a BL course were $293,341, whereas the traditional learning was higher costs at $482,351 [34]. Thus, using BL to teach BLS may decrease the financial burden on the healthcare system by reducing the cost of facilities.

Finally, the challenges of emergency crises such as the COVID-19 pandemic, which restricted face-to-face meeting in all life events, emphasize the need for another educational module. Blended learning or distance learning (DL) has provided an excellent result in dealing with trauma patients and as an alternative to traditional approach in this pandemic [35,36]. Blended learning is one of the methods that proved its efficacy and overcame this obstacle by supporting distancing and decreasing social contact as well as minimizing the crowdedness of participants, instructors, and administrators in the classrooms and decreasing the risk of infection in general and COVID-19 in specific. A literature review was conducted to determine the efficacy and knowledge of nursing students in performing CPR procedures by implementing a BL program through online learning platforms during the COVID-19 pandemic, they highly recommended BL during the COVID-19 pandemic to teach BLS due its effectiveness of CPR that increases the efficacy and knowledge of nursing students [37].

### 4.1 Limitations and future research

Some limitations need to be addressed. The small number of included papers included in the study was the primary drawback of this systematic review. The fact that randomized controlled trials were used in all the selected papers, nevertheless, gives a substantial value because it is an essential module and the cornerstone of the integrated learning approach. Additionally, several studies have shown that nursing and other healthcare professionals were not widely well trained and followed-up to master the BLS knowledge and skills. Furthermore, among the 11 included RCTs, there was heterogeneity in many aspects including the kind of participants, settings, nature, and time of the BL module. Some included laypeople, others nursing or medical students, and some included high school students. Finally, no study was found to examine the impact of BL of BLS on patient outcomes. Therefore, we suggest researchers conduct RCTs to assess the effectiveness of BL on various aspects of patients' outcomes in future. Robust designs and large sample size should be considered when conducting future research. In addition, we recommend to housing the clinical workflow of health care professionals, clinical decision system to judge the quality of using such advanced technologies in improving both knowledge and skills of blended method of learning which may eventually reflected positively on patient outcome and healthcare system.

### 4.2 Implications for future practice and policy

The BL is a strategy that has been demonstrated to increase the knowledge and skills of healthcare professionals, improving high-quality results for cardiovascular patients. Additionally, encouraging healthcare professionals to use the blended form of training will boost their attitude, self-assurance, and competency. For policymakers, applying specific guidelines according to the latest AHA international guidelines to be parallel with the applications of BL will improve the quality indicators, patient outcomes, length of stay, and thus the quality of care.

## 5 Conclusion

Basic life support is an essential component in increasing the survival rate among cardiac arrest victims. There are many methods to teach BLS, two of which are the face-to-face and the BL. The latter one surge in the educational strategy in general and in BLS in more specific. Blended learning of BLS improves and maintains CPR performance accuracy and ability toward patients’ outcomes. The pandemic of COVID-19 gives new learning strategies (lessons) to the educational system including the health sector to adopt another method of learning rather than face-to-face alone. Updated evidence-based research stated that BL of BLS is at least not-inferior to traditional face-to-face regarding skills and knowledge. Hence, embracing this approach to education BLS is pivotal, which may reduce time, resources, and financial burden on the healthcare system.

## Funding

This systematic review has not received any funds.

## Ethics declarations

Review and/or approval by an ethics committee was not needed for this study because [No contact with participants or records, it is a systematic review of published and available work]. Informed consent was not required for this study because [No contact with participants or records, it is a systematic review of published and available work].

## Data availability statement

Data will be made available on request.

## CRediT authorship contribution statement

**Ashraf Jehad Abuejheisheh:** Conceptualization, Data curation, Formal analysis, Investigation, Methodology, Project administration, Resources, Software, Supervision, Validation, Visualization, Writing – original draft, Writing – review & editing. **Jafar Alasad Alshraideh:** Data curation, Writing – original draft. **Nawwaf Amro:** Formal analysis, Software, Validation. **Salam Bani Hani:** Formal analysis, Supervision, Visualization. **Muhamamd Waleed Darawad:** Investigation, Visualization, Writing – review & editing.

## Declaration of competing interest

The authors declare that they have no known competing financial interests or personal relationships that could have appeared to influence the work reported in this paper.
